# Loss of Leptin-Induced Modulation of Hippocampal Synaptic Trasmission and Signal Transduction in High-Fat Diet-Fed Mice

**DOI:** 10.3389/fncel.2017.00225

**Published:** 2017-07-28

**Authors:** Marco Mainardi, Matteo Spinelli, Federico Scala, Andrea Mattera, Salvatore Fusco, Marcello D’Ascenzo, Claudio Grassi

**Affiliations:** Institute of Human Physiology, Medical School, Universita Cattolica del Sacro Cuore Rome, Italy

**Keywords:** patch-clamp, STAT-3, SOCS-3, ObR, CA1, Schaffer collateral

## Abstract

Hippocampal plasticity is triggered by a variety of stimuli including sensory inputs, neurotrophins and inflammation. Leptin, whose primary function is to regulate food intake and energy expenditure, has been recently shown to affect hippocampal neurogenesis and plasticity. Interestingly, mice fed a high-fat diet (HFD) exhibit impaired hippocampal function, but the underlying mechanisms are poorly understood. To address this issue, we compared leptin responsiveness of hippocampal neurons in control and HFD-fed mice by combining single-cell electrophysiology and biochemical assays. We found that leptin modulated spontaneous and evoked synaptic transmission in control, but not HFD, mice. This functional impairment was paralleled by blunted activation of STAT-3, one of the key signal transduction pathways controlled by the fully functional isoform of the leptin receptor, ObRb. In addition, SOCS-3 expression was non-responsive to leptin, indicating that modulation of negative feedback impinging on ObRb was also altered. Our results advance the understanding of leptin action on hippocampal plasticity and, more importantly, suggest that leptin resistance is a key determinant of hippocampal dysfunction associated with hypercaloric diet.

## Introduction

The hippocampus plays a central role in learning and in the consolidation of new memories. This function is accomplished through plasticity at both functional (i.e., synaptic transmission and circuit activity) and structural (i.e., synapse remodeling and neurogenesis) levels. Hippocampal plasticity is affected by a variety of modulators, including neurotrophins (Aicardi et al., 2004; Conner et al., 2009), inflammatory mediators (Habbas et al., 2015) and other extracellular molecules (e.g., extracellular matrix components; Dityatev et al., 2010). More recently, soluble factors involved in metabolic signaling have been proposed to markedly affect hippocampal plasticity (Mainardi et al., 2013, 2015). Among these, leptin is a notable example. It was first identified as the messenger conveying signals about the status of long-term energy stores (i.e., white adipose tissue) and satiation to the basal hypothalamus (Friedman and Halaas, 1998). However, expression of ObRb, the fully functional isoform of leptin receptor, is not confined to cerebral nuclei involved in metabolic homeostasis. Indeed, the hippocampus displays a strikingly high level of ObRb expression (Scott et al., 2009; Caron et al., 2010). Accordingly, leptin has been shown to modulate neurogenesis, dendritic spine generation and synaptic transmission by activating the STAT-3, PI3 kinase and ERK signaling pathways (Coppari and Bjørbæk, 2012; Harvey, 2013), thus exerting a neurotrophin-like action on the hippocampus. Facilitation of plasticity is, in turn, correlated to improved performance in behavioral tasks involving learning and memory (Oomura et al., 2006; Morrison, 2009; Mainardi et al., 2015). Moreover, leptin has neuroprotective effects in experimental models of stroke (Zhang and Chen, 2008) and in transgenic mice reproducing the key features of Alzheimer’s disease (AD; Pérez-González et al., 2014).

The delicate balance regulating the action of leptin is altered in rodents fed with a high-fat diet (HFD), which mimics a hypercaloric Western-style diet (de Git and Adan, 2015). This dietary regimen is responsible for the development of insulin and leptin resistance, thus leading to obesity and type II diabetes (Morrison et al., 2009; Balland and Cowley, 2015). In addition to these metabolic consequences, several reports point to impaired performance in learning and memory tasks involving the hippocampus, such as the Morris water maze (Boitard et al., 2014) and the novel object recognition test (Heyward et al., 2012). Consistently, long-term depression (LTD) and long-term potentiation (LTP) evoked at CA1 synapses are impaired (Valladolid-Acebes et al., 2012; Hao et al., 2016). Accumulating clinical evidence also points to a significant negative effect of hypercaloric diet and obesity on cognitive performance (Wang et al., 2016). To this regard, obesity is now considered a risk factor for AD (McGuire and Ishii, 2016).

Despite the importance of the topic, the consequences of HFD on hippocampal function have not been exhaustively investigated. In the present work, we focused our attention on the effects of leptin on the modulation of spontaneous and evoked synaptic transmission at the Schaffer Collateral (SC)-CA1 synapse. We found that HFD-fed mice failed to display the enhancement of synaptic transmission that leptin induced in mice fed a standard diet (SD). Parallel molecular analyses revealed that the signaling pathways downstream ObRb activation were impaired in HFD mice. Specifically, we found decreased STAT-3 activation, and increased basal SOCS-3 expression that was no more responsive to leptin, thus indicating an impairment of this negative feedback impinging on ObRb.

Our data support the view that leptin affects hippocampal function and provide evidence that a HFD causes hippocampal leptin resistance. These results can have important consequences for the understanding the basis of impaired cognitive performance in both animals and humans subjected to a hypercaloric diet.

## Materials and Methods

### Animals and Diet

Male C57BL/6 mice were weaned at postnatal (P) day 21 and fed *ad libitum* with either a HFD (23% proteins; 42% carbohydrates, namely 28% starch, 9% sucrose, 5% maltodextrin; 34% fats; 60% fat caloric content; PF4051/D, Mucedola, Italy) or a standard chow diet (SD; 18.5% proteins; 46% carbohydrates, namely 42% starch, 4% sucrose; 3% fats; 6.55% fat caloric content; 4RF21, Mucedola) for 8 weeks, then used for tissue collection or electrophysiology experiments. The HFD regimen resulted in overweight and hyperglycemia (Supplementary Figure S1). Mice were housed under a 12-h light-dark cycle at constant room temperature (22°C). All animal procedures were approved by the Ethics Committee of Catholic University and were fully compliant with Italian (Ministry of Health guidelines, Legislative Decree No. 26/2014) and European Union (Directive No. 2010/63/UE) laws on animal research. The experiments were carried out in strict accordance with the approved guidelines.

### Electrophysiology and Data Analysis

For the preparation of brain slices, we followed the protocol described in Curcio et al. (2013), with minor modifications. Animals were euthanized by cervical dislocation and decapitated. The brains were rapidly removed and placed in ice-cold, sucrose-based cutting solution containing the following (in mM): TRIS-HCl 72, TRIZMA base 18, NaH_2_PO_4_ 1.2, NaHCO_3_ 30, KCl 2.5, glucose 25, HEPES 20, MgSO_4_ 10, Na-pyruvate 3, ascorbic acid 5, CaCl_2_ 0.5, sucrose 20. Slices (300 μm thick) were cut on a vibratome (VT1200S; Leica Microsystems, Germany) and immediately transferred to an incubation chamber held at 32°C and filled with a recovery solution containing (in mM): TRIS-HCl 72, TRIZMA base 18, NaH_2_PO_4_ 1.2, NaHCO_3_ 25, KCl 2.5, glucose 25, HEPES 20, MgSO_4_ 10, Na-pyruvate 3, ascorbic acid 5, CaCl_2_ 0.5, sucrose 20. After 30 min, slices were transferred to a second incubation chamber held at 32°C and filled with artificial cerebrospinal fluid (aCSF) containing the following (in mM): NaCl 124, KCl 3.2, NaH_2_PO_4_ 1.2, MgCl_2_ 1, CaCl_2_ 2, NaHCO_3_ 26 and glucose 10, pH 7.4. During incubations, the chambers were continuously bubbled with 95% O_2_/5% CO_2_. Finally, slices were equilibrated at RT for at least 45 min. For electrophysiological recordings, slices were transferred to a submerged recording chamber constantly perfused with heated aCSF (32°C) and bubbled with 95% O_2_/5% CO_2_. Neurons of the CA1 area were visualized under DIC infrared illumination. Stimulation of the SC was obtained by means of a current stimulus isolator (WPI, Worcester, MA, USA), connected to a bipolar concentric stimulating electrode (FHC, Bowdoin, ME, USA) which was positioned in contact with the SC pathway. Patch pipettes had a resistance of 4–6 MΩ when filled with an internal solution containing (in mM): K-gluconate 145, MgCl_2_ 2, HEPES 10, EGTA 0.1, Na-ATP 2.5, Na-GTP 0.25, phosphocreatine 5, pH adjusted to 7.2 with KOH. For AMPA/NMDA ratio experiments, the internal solution contained (in mM): CsCH_3_SO_3_ 135, HEPES 10, NaCl 8, EGTA 0.25, MgCl_2_ 2, Mg-ATP 4. Na-GTP 0.3, phosphocreatine 5, pH adjusted to 7.3 with NaOH. For rectification index measurements, 0.1 mM spermine was added to this solution. All the chemicals were purchased from Sigma-Aldrich (Germany) and all the blockers from Tocris (USA), unless otherwise stated. After establishing a gigaseal, the patch was broken by applying negative pressure to achieve a whole-cell configuration. A series resistance lower than 15 MΩ was considered acceptable, and monitored constantly throughout the entire recording. For evoked and spontaneous Excitatory Postsynaptic Currents (EPSC) measurements, neurons were held at −70 mV and electrical stimuli were delivered to the SC. First, the input-output relationship was assessed in order to find the maximal response amplitude. Subsequent measurements were performed using a stimulation that yielded 30% of the maximal response. Paired-pulse facilitation (PPF) was assessed by delivering pairs of stimuli at different interstimulus intervals (ISIs; 20, 50, 100, 200 ms), repeated at 0.05 Hz. To obtain the AMPA/NMDA currents ratio, stimuli of identical amplitude were delivered at holding potentials of −70 and +40 mV, as described in Ahmad et al. (2012), with a frequency of 0.05 Hz; 50 μM picrotoxin (PTX) was added to the bath. Using this protocol, measurement of both currents with no need to use pharmacological blockers could be obtained, thus allowing their testing before and after leptin application. The rectification index was calculated by recording the evoked EPSCs at −60, −40, 0, +40 and +60 mV holding potentials in the presence of 50 μM of the NMDA receptor blocker D-2-amino-5-phosphonovalerate (D-AP5) and 50 μM PTX, then by computing the ratio (slope of the −60 to 0 mV regression line)/(slope of the 0 to +60 mV regression line). The identity of evoked EPSCs was confirmed at the end of the recordings by adding 10 μM of the selective AMPA receptor blocker 2,3-Dioxo-6-nitro-1,2,3,4-tetrahydrobenzo[*f*]quinoxaline-7-sulfonamide (NBQX) to the bath. For miniature EPSCs (mEPSCs) measurements, 0.5 μM tetrodotoxin and 50 μM PTX were applied to the bath. After measuring the baseline parameters (i.e., maximum AMPA response, AMPA/NMDA ratio, PPF, spontaneous EPSCs (sEPSCs) or mEPSCs), murine leptin (Peprotech, UK) was bath-applied at a 50 nM concentration, which has been previously demonstrated to be the minimal dose required to exert a maximal effect (Shanley et al., 2001; Luo et al., 2015). After application, 10 min were allowed and measurements were repeated. In control experiments, vehicle (saline) alone was used. Recordings were performed using a Multiclamp 700B/Digidata 1550A system (Molecular Devices, Sunnyvale, CA, USA) and digitized at a 10,000 Hz sampling frequency. All the electrophysiological recordings were analyzed using the Clampfit 10.6 software (Molecular Devices). AMPA receptor-mediated EPSC amplitude was calculated as the difference between the peak response and the baseline. NMDA receptor-mediated EPSC amplitude was calculated as the amplitude 50 ms after the response onset (Ahmad et al., 2012). For PPF analysis, the ratio between the second and first evoked responses was calculated. For mEPSC and sEPSC frequency analysis, a template was constructed using the “Event detection/create template” function, as described in Ripoli et al. (2013). Then, mEPSCs and sEPSCs were detected using the “Event detection/template search” function; the “template match threshold” was set to 3.5 and the result inspected for false positives. For mEPSC and sEPSC amplitude analysis, all the waveforms detected during a single recording using template analysis were averaged and the amplitude calculated. All the data are normalized as ((response post-leptin application)/(baseline response)) × 100.

### Western Blotting and Co-Immunoprecipitation

For *in vivo* administration, leptin was dissolved in saline and i.p. injected (3 mg/kg), using an adaptation of the protocol described in Mainardi et al. (2010). Mice were fasted for 2 h, injected and hippocampi dissected either 45 min or 2 h post-injection. Tissues were immediately frozen in liquid nitrogen and stored at −80°C until further processing. For protein expression and phosphorylation, tissues were lysed in ice-cold lysis buffer containing (in mM): NaCl 150, Tris-HCl 50 (pH 8), EDTA 2, NaF 1, Na_3_VO_4_ 1, PMSF 1, 1% Triton X-100, 1% SDS, 1X protease inhibitor mixture (Sigma-Aldrich, Germany). After 15 min on ice, the lysates were centrifuged at 22,000 *g*, 4°C to remove debris, and the supernatant quantified for protein content (DC Protein Assay; Bio-Rad, USA). Equal amounts of protein lysates were diluted in 6× Laemmli buffer, boiled and resolved by SDS-PAGE, using either 12% (STAT-3 and SOCS-3 detection) or 7% (ObRb detection) acrylamide gels. Western blotting was performed by incubating primary antibodies overnight at 4°C with gentle rocking and revealed with horseradish peroxidase-conjugated secondary antibodies diluted at 1:5000 (7074 and 7076, Cell Signaling, USA; sc-2020, Santa Cruz, USA). The following primary antibodies were used: rabbit monoclonal anti-^705^Tyr-pSTAT-3 (1:1000, Cell Signaling 9145), mouse monoclonal anti-STAT-3 (1:1000, Cell Signaling 9139), mouse monoclonal anti-ObR (1:1000, Santa Cruz sc-8391), goat polyclonal anti-^1138^Tyr-pObR (1:1000, Santa Cruz sc-16421), rabbit polyclonal anti-^473^Ser-pAkt (1:1000, Cell Signaling 4060), rabbit polyclonal anti-Akt (1:1000, Cell Signaling 9272), rabbit polyclonal anti-SOCS-3 (1:1000, Cell Signaling 2923), mouse monoclonal anti-α-tubulin (1:8000, Sigma-Aldrich T5168), rabbit polyclonal anti-GluR1 (1:1000, Cell Signaling D4N9V), mouse monoclonal anti-GluR2 (1:1000, Millipore MAB391). For co-immunoprecipitation experiments, tissues were lysed in low-detergent buffer containing (in mM): KCl 50, Tris-HCl 50 (pH 8), EDTA 10 and 1% NP-40. Lysates were centrifuged at 22,000 *g* to remove debris and a fraction of the supernatant was used for assessing the quality of the total lysate. Lysates were precleared for 30 min with empty protein G-sepharose 4B beads (Sigma-Aldrich) before being challenged with 1–2 μg of specific antibody (mouse anti-ObRb) or IgG control and fresh protein G matrix. After a 6-h incubation at 4°C with a rotating mixer, protein-G-bound immunocomplexes were collected by centrifugation (22,000 *g*, 1 min) and washed six times with 500 μl of immunoprecipitation buffer. Beads were finally resuspended in 30 μL of 1× Laemmli buffer and boiled. Eluted proteins were subjected to SDS-PAGE and immunoblotting as described above. Blots were imaged and analyzed using an Alliance imaging platform (Uvitec, UK) and the optical densities were normalized as follows: (phosphoprotein “X”)/(total levels of protein “X”)/(α-tubulin) and (total levels of protein “X”)/(α-tubulin). For each experimental replica, data were normalized on the average relative optical density of the control group (i.e., SD-veh).

### Statistical Analyses

Data were analyzed using the SigmaPlot 12 software (Systat, USA). Statistical significance was assessed using one- or two-way analysis of variance (ANOVA-1 and -2, respectively), with factor 1 = diet (i.e., SD or HFD) and factor 2 = treatment (i.e., leptin or vehicle). When a significant interaction was detected, groups were compared using Bonferroni *post hoc* test. For comparisons between SD and HFD groups only, Student’s *t* test was used. Statistically significant differences between mEPSC cumulative distributions were assessed using Kolmogorov-Smirnov test. Data are expressed as mean ± SEM.

## Results

### HFD Mice did Not Exhibit Leptin-Driven Enhancement of Spontaneous Neurotransmitter Release

**Figure 1:**
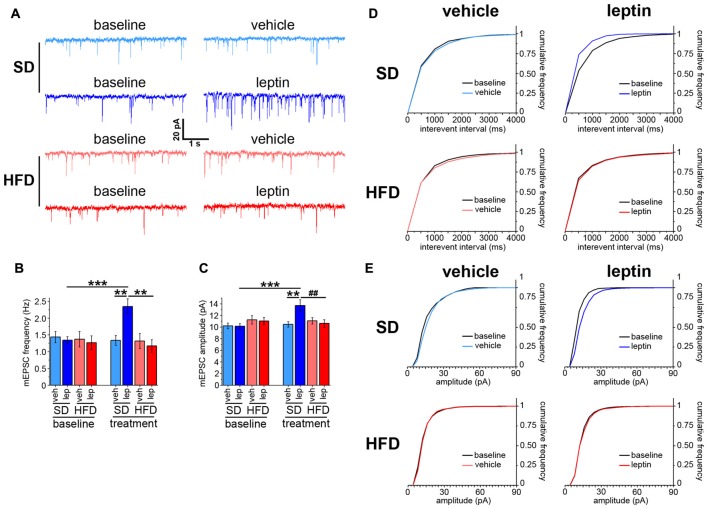
Leptin modulates spontaneous neurotransmitter release in standard diet (SD), but not in high-fat diet (HFD), mice. **(A)** Representative traces showing miniature excitatory postsynaptic current (mEPSC) recordings in SD and HFD mice treated with vehicle or leptin; both baseline and post-treatment (i.e., vehicle or leptin) recordings, corresponding to the same cell, are shown. **(B)** mEPSC frequency is increased in SD mice in response to leptin application; in contrast, HFD mice are resistant to this effect (SD-veh *n* = 13, SD-lep *n* = 11, HFD-veh *n* = 11, HFD-lep *n* = 14; analysis of variance (ANOVA)-2 diet × treatment interaction *p* = 0.009, followed by Bonferroni *post hoc* test, **p = 0.002, ***p < 0.001). **(C)** SD mice display higher mEPSC amplitude in response to leptin application, whereas HFD mice fail to respond to the treatment (SD-veh *n* = 13, SD-lep *n* = 11, HFD-veh *n* = 11, HFD-lep *n* = 14; ANOVA-2, diet × treatment interaction, *p* = 0.007, followed by Bonferroni *post hoc* test, ^##^*p* = 0.006, **p = 0.005, ***p < 0.001). **(D)** Cumulative frequency distributions for mEPSC interevent intervals, showing a leftwards shift for SD-lep mice (blue curve, upper right panel, Kolmogorov-Smirnov test, *p* = 0.035) in contrast to no shift in HFD-lep mice (red curve, lower right panel, Kolmogorov-Smirnov test, *p* = 0.95). **(E)** Cumulative frequency distributions for mEPSC amplitude, showing a rightwards shift for SD-lep mice (blue curve, upper right panel, Kolmogorov-Smirnov test, *p* = 0.01) in contrast to no shift in HFD-lep mice (red curve, lower right panel, Kolmogorov-Smirnov test, *p* = 0.42).

Administration of leptin to cultured hippocampal neurons has been reported to increase both frequency and amplitude of mEPSCs (Dhar et al., 2014). First, we checked whether a similar effect was observed in hippocampus brain slices obtained from adult mice fed a SD. Voltage-clamp experiments revealed that administration of leptin increased the frequency of mEPSCs from 1.34 ± 0.11 Hz to 2.35 ± 0.23 Hz (Figures 1A,B). Accordingly, the cumulative frequency distribution for interevent intervals was shifted to the left after treatment with leptin (Figure 1D). A significant enhancement of mEPSC amplitude, from 10.15 ± 0.43 pA to 13.68 ± 0.99 pA, was also observed (Figures 1A,C). Consistent with this finding, the cumulative frequency distribution of event amplitudes was shifted to the right after leptin treatment (Figure 1E). A different picture emerged when we performed similar measurements on hippocampal slices obtained from mice fed a HFD. Indeed, leptin failed to alter both mEPSC frequency and amplitude, which amounted to 1.17 ± 0.19 Hz (baseline 1.27 ± 0.21 Hz) and 10.62 ± 0.65 pA (baseline 11.03 ± 0.63 pA), respectively (Figures 1A–C). In keeping with this, the corresponding cumulative frequency distributions before and after leptin treatment were superimposable (Figures 1D,E). The baseline frequencies of mEPSCs were not significantly different among the four experimental groups (SD-veh, 1.44 ± 0.17 Hz; SD-lep, 1.34 ± 0.11 Hz; HFD-veh, 1.37 ± 0.23 Hz; HFD-lep, 1.27 ± 0.21 Hz; ANOVA-2, *p*_diet_ = 0.713, *p*_treatment_ = 0.589, *p*_interaction_ = 0.974). The same observation emerged from analysis of mEPSC amplitudes (SD-veh, 10.20 ± 0.50 pA; SD-lep, 10.15 ± 0.43 pA; HFD-veh, 11.23 ± 0.72 pA; HFD-lep, 11.03 ± 0.63 pA; ANOVA-2, *p*_diet_ = 0.100, *p*_treatment_ = 0.825, *p*_interaction_ = 0.896). To better address the point of neurotransmitter release, we also recorded sEPSCs, i.e., by omitting PTX and tetrodotoxin from the bath (see “Materials and Methods” Section). In the SD-lep group, leptin caused the sEPSC frequency to rise from 1.73 ± 0.18 Hz to 2.57 ± 0.27 Hz, whereas in the HFD-lep group it remained to 1.69 ± 0.24 Hz (baseline 1.88 ± 0.25 Hz; Supplementary Figures S2A,B). On the other hand, no significant effects were found on sEPSC amplitude in both SD-lep and HFD-lep groups (SD-lep, baseline 13.76 ± 1.40 pA, post-leptin 13.74 ± 1.44 pA; HFD-lep, baseline 11.74 ± 0.79 pA, post-leptin 11.05 ± 0.88 pA; ANOVA-2, *p*_diet_ = 0.523, *p*_treatment_ = 0.750, *p*_interaction_ = 0.989). Also in this case, the baseline frequencies (SD-veh, 1.71 ± 0.18 Hz; SD-lep, 1.73 ± 0.18 Hz; HFD-veh, 1.73 ± 0.23 Hz; HFD-lep, 1.88 ± 0.25 Hz; ANOVA-2, *p*_diet_ = 0.585, *p*_treatment_ = 0.828, *p*_interaction_ = 0.589) and amplitudes (SD-veh, 13.22 ± 1.53 pA; SD-lep, 13.76 ± 1.40 pA; HFD-veh, 12.04 ± 1.00 pA; HFD-lep, 11.74 ± 0.79 pA; ANOVA-2, *p*_diet_ = 0.358, *p*_treatment_ = 0.863, *p*_interaction_ = 0.958) were not significantly different among the four groups. The trend towards a reduced sEPSC amplitude in HFD mice, compared to SD mice, was also non-significant (Supplementary Figures S2A,C).

These data show that leptin affects basal synaptic transmission at CA3-CA1 synapses in adult SD mice, and that this effect is lost in HFD mice.

### HFD Mice Failed to Potentiate Evoked EPSCs and PPF in Response to Leptin Administration

**Figure 2:**
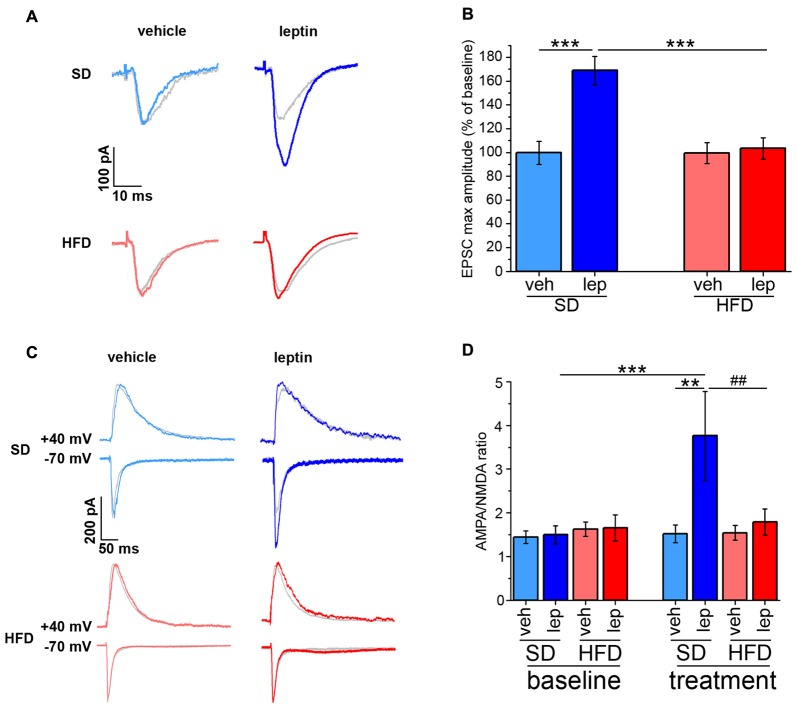
HFD mice are resistant to potentiation of AMPA receptor-mediated evoked excitatory postsynaptic currents (EPSCs) by leptin. **(A)** Representative traces showing the amplitude of AMPA receptor-mediated EPSCs before and after application of either vehicle or leptin. The post-treatment response is superimposed to the baseline response of the same cell, depicted in gray. **(B)** Leptin induces potentiation of AMPA receptor-mediated evoked EPSCs is SD mice; this effect is completely abolished in HFD mice (SD-veh *n* = 11, SD-lep *n* = 14, HFD-veh *n* = 12, HFD-lep *n* = 16; ANOVA-2, diet × treatment interaction, *p* = 0.002, followed by Bonferroni *post hoc* test, ***p < 0.001). **(C)** Representative traces showing AMPA and NMDA receptor-mediated evoked EPSCs (−70 and +40 mV holding potentials, respectively) before and after application of either vehicle or leptin. Baseline responses are depicted in gray and are superimposed to the post-treatment response of the same cell. **(D)** AMPA/NMDA curents ratio of SD mice is enhanced by leptin, whereas HFD mice fail to display this effect (SD-veh *n* = 10, SD-lep *n* = 11, HFD-veh *n* = 12, HFD-lep *n* = 10; ANOVA-2, diet × treatment interaction, *p* = 0.017, followed by Bonferroni *post hoc* test, ^##^*p* = 0.008, **p = 0.003, ***p < 0.001).

The amplitude of currents evoked by stimulation of SC afferents to CA1 neurons is potentiated by leptin application, thus resulting in a long-lasting, LTP-like effect (Moult and Harvey, 2011). By performing voltage-clamp EPSC recordings, we confirmed that this effect was also observed in our experimental conditions. Indeed, leptin application to slices obtained from SD mice induced a potentiation of EPSC amplitude in CA1 neurons reaching 169 ± 12% of baseline (Figures 2A,B). However, no significant changes in EPSC amplitude (103 ± 9% of baseline) were induced by leptin in brain slices of HFD mice (Figures 2A,B).

Expression of synaptic plasticity involves changes in the ratio between AMPA receptor- and NMDA receptor-mediated EPSCs (Ahmad et al., 2012). To investigate whether leptin affected this parameter, we performed EPSC recordings by alternatively switching between −70 mV and +40 mV holding potentials (see “Materials and Methods” Section). In slices obtained from SD mice, CA1 neurons responded to leptin with an increase in the AMPA/NMDA EPSC ratio from 1.59 ± 0.21 to 3.76 ± 1.02 (Figures 2C,D). Instead, in slices obtained from HFD mice, the AMPA/NMDA EPSC ratio was not significantly affected by leptin (baseline, 1.65 ± 0.30, post-leptin, 1.79 ± 0.30; Figures 2C,D). This effect was a consequence of the potentiation of AMPA receptor-mediated EPSCs in SD mice only (SD-veh, 105.11 ± 7.37%; SD-lep, 170.12 ± 13.18%; HFD-veh 107.51 ± 14.80%; HFD-lep, 111.29 ± 10.76% of baseline value; ANOVA-2, diet × treatment interaction, *p* = 0.013, followed by Bonferroni *post hoc* test, SD-lep vs. SD-veh, *p* < 0.001, SD-lep vs. HFD-lep, *p* = 0.002), whereas no significant difference in NMDA receptor-mediated EPSCs before and after leptin application was detected (SD-veh, 82.44 ± 8.182%; SD-lep, 91.19 ± 31.58%; HFD-lep, 89.97 ± 10.33%; HFD-lep, 98.35 ± 14.59% of baseline value; ANOVA-2, *p*_diet_ = 0.699, *p*_treatment_ = 0.652, *p*_interaction_ = 0.992). The basal AMPA/NMDA ratio was not significantly different among the four experimental groups (SD-veh, 1.44 ± 0.15; SD-lep, 1.50 ± 0.21; HFD-veh, 1.62 ± 0.16; HFD-lep, 1.65 ± 0.30; ANOVA-2, *p*_diet_ = 0.416, *p*_treatment_ = 0.829, *p*_interaction_ = 0.948). Addition of the selective AMPA blocker NBQX abolished the inward evoked currents described above, thus confirming the identity of the recorded EPSCs (Supplementary Figure S3).

**Figure 3:**
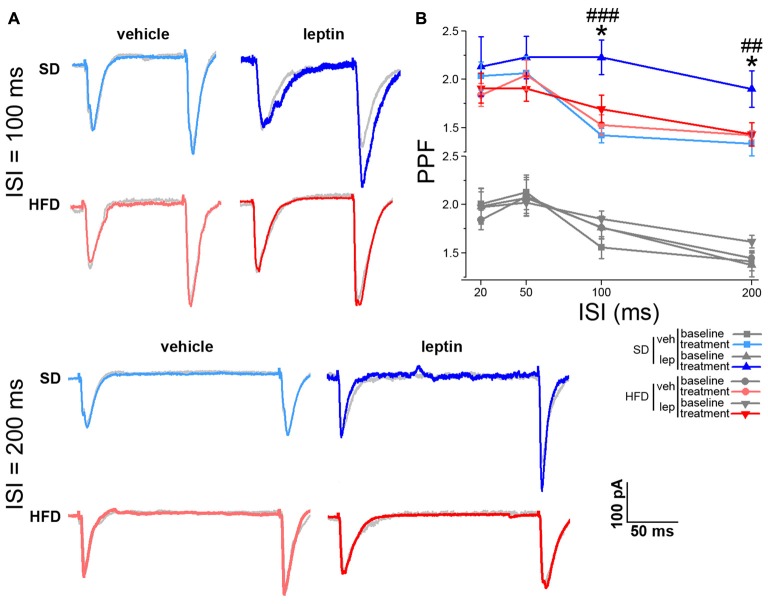
HFD mice are resistant to leptin-mediated increase in paired-pulse facilitation (PPF). **(A)** Representative traces showing EPSCs evoked by pair of stimuli delivered to the Schaffer Collateral (SC) at 100 ms (upper panel) and 200 ms (lower panel) interstimulus intervals (ISIs). The post-treatment response is superimposed to the baseline response of the same cell, depicted in gray. **(B)** Leptin treatment increases PPF at 100 ms (SD-veh *n* = 10, SD-lep *n* = 10, HFD-veh *n* = 11, HFD-lep *n* = 14; ANOVA-2, diet × treatment interaction, *p* = 0.022, followed by Bonferroni *post hoc* test, SD-lep vs. SD-veh, ^###^*p* < 0.001, SD-lep vs. HFD-lep, *p = 0.013) and 200 ms (SD-veh *n* = 10, SD-lep *n* = 10, HFD-veh *n* = 11, HFD-lep *n* = 14; ANOVA-2, diet × treatment interaction, *p* = 0.009, followed by Bonferroni *post hoc* test, SD-lep vs. SD-veh, ^##^*p* = 0.005, SD-lep vs. HFD-lep, *p = 0.02) ISI in SD mice; in contrast, HFD mice are resistant to the action of leptin on PPF. Baseline and post-treatment plots have been separated to increase clarity. Both plots are shown on the same range, with no magnification.

The effects on synaptic plasticity and transmission prompted us to address whether leptin could also affect short-term plasticity of the SC-CA1 pathway. We approached this point using the paired-pulse protocol, by delivering electrical stimuli to the SC at ISIs of 20, 50, 100 and 200 ms (see “Materials and Methods” Section). When pairs of stimuli at low ISIs (i.e., 20 and 50 ms) were delivered to CA1 neurons of SD mice, we did not observe any significant effect on PPF in response to leptin application (Supplementary Figure S4). This finding is in line with previous literature reports (Moult et al., 2009). Interestingly, a significant increase in PPF was found at longer ISIs (from 1.76 ± 0.12 to 2.23 ± 0.18, and from 1.38 ± 0.12 to 1.90 ± 0.19 for 100 and 200 ms ISI, respectively, Figure 3). Also in this case, in slices from HFD mice leptin failed to elicit any significant change in PPF at all ISIs we examined (from 1.85 ± 0.08 to 1.70 ± 0.14, and from 1.62 ± 0.07 to 1.43 ± 1.12 for 100 and 200 ms ISI, respectively; Figure 3). The basal PPF values did not differ among the four experimental groups for both 100 ms (SD-veh, 1.56 ± 0.11; SD-lep, 1.76 ± 0.12; HFD-veh, 1.76 ± 0.12; HFD-lep, 1.85 ± 0.08; ANOVA-2, *p*_diet_ = 0.178, *p*_treatment_ = 0.177, *p*_interaction_ = 0.588) and 200 ms (SD-veh, 1.41 ± 0.1; SD-lep, 1.377 ± 0.12; HFD-veh, 1.45 ± 0.08; HFD-lep, 1.62 ± 0.07; ANOVA-2, *p*_diet_ = 0.129, *p*_treatment_ = 0.458, *p*_interaction_ = 0.249) ISI. These effects do not appear to be due to a difference in the AMPA rectification index, which was 1.36 ± 0.24 in SD mice and 1.33 ± 0.21 in HFD mice (Supplementary Figures 5A–C). In keeping with this, the GluR1 and GluR2 AMPA receptor subunits levels were not significantly different between SD and HFD mice (Supplementary Figures 5D–F).

Taken together, these results show that leptin is able to modulate both spontaneous and evoked neurotransmission at the SC-CA1 pathway. Notably, this can be observed only in mice fed a SD, whereas a HFD regimen results in hippocampal resistance to these functional effects of leptin.

### Blunted Activation of STAT-3 by Leptin in HFD Mice

**Figure 4:**
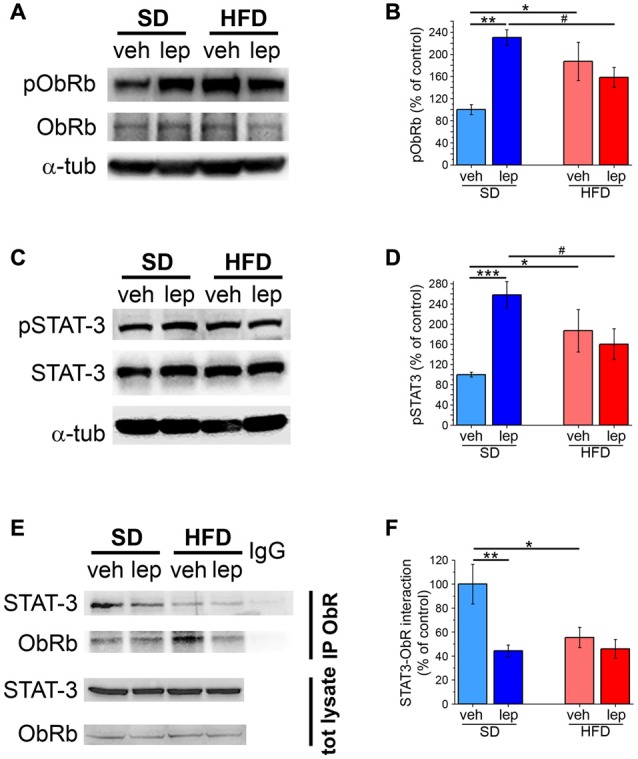
Activation of ObRb and STAT-3 is impaired in HFD mice. **(A)** Representative images showing immunoblottings for pObRb, ObRb and α-tubulin. **(B)** Quantification of pObRb activation (SD-veh *n* = 4, SD-lep *n* = 4, HFD-veh *n* = 4, HFD-lep *n* = 4; ANOVA-2, diet × treatment interaction, *p* = 0.004, followed by Bonferroni *post hoc* test, SD-lep vs. SD-veh, **p = 0.002, SD-veh vs. HFD-veh, *p = 0.019, SD-lep vs. HFD-lep, ^#^*p* = 0.046). **(C)** Representative images showing immunoblottings for pSTAT-3, STAT-3 and α-tubulin. **(D)** Quantification of pSTAT-3 activation (SD-veh *n* = 5, SD-lep *n* = 6, HFD-veh *n* = 5, HFD-lep *n* = 5; ANOVA-2, diet × treatment interaction, *p* = 0.003, followed by Bonferroni *post hoc* test, SD-lep vs. SD-veh, ***p < 0.001, SD-lep vs. HFD-lep, *p = 0.019; SD-veh vs. HFD-veh, ^#^*p* = 0.029). **(E)** Representative images showing immunoblottings for STAT-3 after immunoprecipitation using an anti-ObRb antibody and for the total protein lysates. **(F)** Quantification of STAT-3-ObRb interaction from immunoprecipitates (SD-veh *n* = 5, SD-lep *n* = 5, HFD-veh *n* = 4, HFD-lep *n* = 6; ANOVA-2, diet × treatment interaction, *p* = 0.043, followed by Bonferroni *post hoc* test, SD-lep vs. SD-veh, **p = 0.002; SD-veh vs. HFD-veh, *p = 0.011).

Signal transduction is instrumental to the actuation of leptin effects on cellular functions. Among the pathways controlled by the leptin receptor, ObRb, a paramount role is played by STAT-3 (Plum et al., 2006; Mainardi et al., 2010; Nicolas et al., 2013). Specifically, phosphorylation of ObRb at ^1138^Tyr results in phosphorylation of STAT-3 at ^705^Tyr. First, we found that SD mice exhibited increased levels of ^1138^Tyr-pObRb 45 min after leptin injection (231 ± 14%; Figures 4A,B). In HFD mice, basal phosphorylation of ObRb was increased and was unresponsive to modulation by leptin (HFD-veh, 187 ± 35%, HFD-lep, 158 ± 18%; Figures 4A,B). Total levels of ObRb were not significantly different among the four experimental groups (Figure 4A and Supplementary Figure S6A).

Then, we analyzed STAT-3 phosphorylation on ^705^Tyr, which in SD-lep mice was 258 ± 26% of SD-veh controls 2 h after leptin injection (Figures 4C,D). In HFD mice (HFD-veh group), we observed a significant increase in basal activation of STAT-3, whose phosphorylation was 187 ± 42% of SD-veh mice (Figures 4C,D). Moreover, in HFD mice STAT-3 activation was not significantly affected by leptin injection (HFD-lep, 161 ± 30% vs. HFD-veh, 187 ± 42%; Figures 4D,E). Notably, no significant differences in the overall expression levels of STAT-3 were found among the four experimental groups (Figure 4C and Supplementary Figure S6B). We observed an analogous trend also for Akt, whose phosphorylation is also modulated by ObRb (Valladolid-Acebes et al., 2012; Gavello et al., 2016). Indeed, in SD-lep mice, Akt phosphorylation on ^473^Ser was increased by leptin to 217 ± 40% of SD-veh controls (Supplementary Figures S7A,B). On the other hand, HFD-veh mice displayed a significantly higher basal pAkt level, which was not responsive to leptin (HFD-veh, 153 ± 11%, HFD-lep 148 ± 16%; Supplementary Figures S7A,B). The total levels of Akt were not significantly different among groups (Supplementary Figures S7A,C). Interestingly, loss of STAT-3 activation in HFD mice could be overcome by injecting an increased dose (5 mg/kg) of leptin, which led to a significant increase in the levels of pSTAT-3 (239 ± 43% of HFD-veh; Supplementary Figure S8).

STAT-3 activation requires its interaction with phosphorylated ObR and its subsequent detachment from the receptor (Plum et al., 2006). We analyzed this interaction by means of co-immunoprecipitation experiments. First, basal association between STAT-3 and ObRb was significantly lower in HFD mice than in SD mice (SD-veh, 100 ± 17%; HFD-veh, 55 ± 8%; Figures 4E,F). In SD-lep mice, STAT-3-ObRb interaction dropped to 44 ± 5% of the baseline value observed in SD-veh mice. On the other hand, in HFD mice, STAT-3-ObRb interaction was virtually insensitive to leptin and, in the HFD-lep group, remained to 46 ± 8% of the baseline value observed in SD-veh mice, which was not significantly different from the value of the HFD-veh group (Figures 4E,F).

### Increased Inhibition of Leptin Signaling in HFD Mice

**Figure 5:**
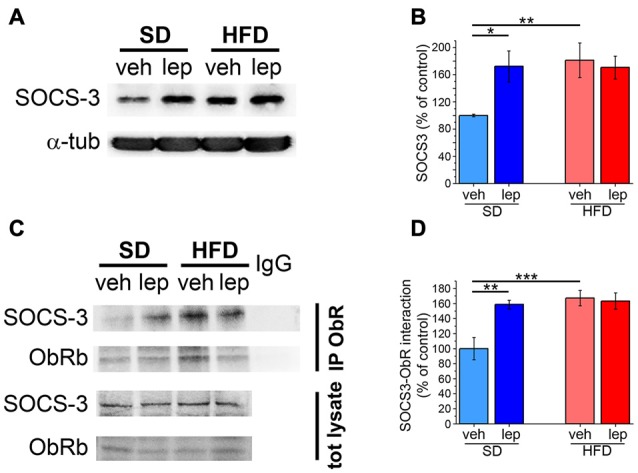
The SOCS-3 inhibitory pathway is altered in HFD mice. **(A)** Representative images showing immunoblottings for SOCS3 and α-tubulin. **(B)** Quantification of SOCS-3 levels (SD-veh *n* = 5, SD-lep *n* = 5, HFD-veh *n* = 5, HFD-lep *n* = 5; ANOVA-2, diet × treatment interaction, *p* = 0.045, followed by Bonferroni *post hoc* test, SD-lep vs. SD-veh, *p = 0.016, SD-veh vs. HFD-veh, **p = 0.008). **(C)** Representative images showing immunoblottings for SOCS-3 after immunoprecipitation using an anti-ObRb antibody and for the total protein lysates. **(D)** Quantification of SOCS-3-ObRb interaction from immunoprecipitates (SD-veh *n* = 5, SD-lep *n* = 5, HFD-veh *n* = 4, HFD-lep *n* = 6; ANOVA-2, diet × treatment interaction, *p* = 0.012, followed by Bonferroni *post hoc* test, SD-lep vs. SD-veh, **p = 0.002; SD-veh vs. HFD-veh, ***p < 0.001).

A possible mechanism involved in leptin resistance is alteration of negative feedback signals aimed at suppressing ObRb activation (Bjorbaek et al., 1998). To ascertain this hypothesis, we measured the levels of SOCS-3 in the hippocampus of HFD mice. The SD-lep group showed a significant increase in SOCS-3 expression 2 h after leptin injection (172 ± 23%, Figures 5A,B), which was in line with previous literature reports (Banks et al., 2000). Interestingly, in HFD-fed mice basal levels of SOCS-3 were higher than in SD-veh mice (HFD-veh, 181 ± 25%; Figures 5A,B) and they were not significantly affected by leptin injection (HFD-lep, 171 ± 17%; Figures 5A,B). Deactivation of ObRb mainly depends upon its binding by SOCS-3. Co-immunoprecipitation experiments revealed that, in SD mice, leptin injection caused this interaction to rise to 159 ± 6% of baseline (SD-lep vs. SD-veh, Figures 5C,D). In HFD mice, we observed that basal SOCS-3-ObRb association amounted to 167 ± 10% of SD mice and this was insensitive to leptin administration, as we did not find any significant difference between the HFD-lep and HFD-veh groups (Figures 5C,D).

These findings demonstrate that HFD is accompanied by higher activation of the SOCS-3 inhibitory pathway, along with loss of its responsiveness to oscillations in leptin levels.

## Discussion

Synaptic transmission and plasticity are widely considered the cellular substrate of learning and memory. Experimental evidence and clinical studies suggest that diet-dependent metabolic signals have the potential to impact on cognitive functions by modulating synaptic function (Morrison, 2009; Harvey, 2013; Mainardi et al., 2015). In particular, leptin affects structural plasticity, neurogenesis and synaptic activity (Harvey, 2013; Mainardi et al., 2013; Rubin et al., 2015), thus playing a neurotrophin-like action exceeding the well-recognized role as a satiety signal. Notably, leptin induces synaptic plasticity with an age-dependent polarity: LTD is stimulated in juveniles, LTP in adults (Moult and Harvey, 2011). On the other hand, mounting evidence points to an action of the dietary style on cognitive performance. Indeed, rodents fed a HFD display a lower performance in behavioral tasks involving the hippocampus (Heyward et al., 2012; Boitard et al., 2014). Thus, we hypothesized that adult HFD-fed mice could display an impairment in leptin-driven modulation of the functional substrates of learning and memory.

First, the results of our work confirm and lend further support to the effects of leptin on synaptic transmission and plasticity of hippocampal circuits in SD conditions. Indeed, we found that leptin enhances spontaneous neurotransmitter release by increasing sEPSC frequency and both frequency and amplitude of mEPSCs, consistently with data obtained in primary hippocampal cultures (Dhar et al., 2014). Stimulus-evoked synaptic responses of CA1 neurons were also affected, and this is compatible with induction of LTP-like postsynaptic phenomena observed in previous work (Moult and Harvey, 2011). Accordingly, we observed an increase in the AMPA/NMDA current ratio, with no detectable effect on NMDA current amplitude, which is likely due to enhanced AMPA receptor trafficking, as it has been observed in culture (Moult and Harvey, 2011), and in agreement with the role of leptin as an ion channel modulator (Gavello et al., 2016). Moreover, we demonstrate here for the first time an effect on short-term plasticity, namely PPF. Previous evidence showed no effect of leptin on PPF of both excitatory and inhibitory neurotransmission at short ISIs (Moult et al., 2009; Guimond et al., 2014). We confirm this finding, using 20 and 50 ms ISIs. However, when the ISI was increased to 100 or 200 ms, we detected a clear increase of PPF, an effect that is usually correlated with a presynaptic site of action (Nicoll and Malenka, 1999). On the other hand, enhanced PPF can also have a postsynaptic origin, as it has been demonstrated to be a hallmark feature of EPSCs mediated by GluR2-lacking AMPA receptors (Liu and Zukin, 2007; Savtchouk and Liu, 2011). Insertion of GluR2-lacking AMPA receptors has indeed been demonstrated at SC-CA1 synapses in response to leptin application (Moult et al., 2010). It is also worth considering that modulation of PPF selectively at longer ISIs has been demonstrated in various experimental models and may point to an effect on GABA_B_ receptor activity (Tsai and Leung, 2006; Gengler et al., 2010). In addition, hypothalamic GABAergic neurons are one of the most relevant targets of leptin (Vong et al., 2011; Lee et al., 2015). The existence of an analogous mechanism in the hippocampus deserves further investigation in the future. Taken together, our electrophysiological data demonstrate that leptin potentiates spontaneous and evoked neurotransmission by acting at both presynaptic and postsynaptic levels. This dual site of action on the SC-CA1 pathway is in agreement with the pattern of ObR expression in the mouse hippocampus, which is widespread, but peaks in the CA3 area (Caron et al., 2010; Wang et al., 2015).

The key finding of our patch-clamp experiments is that, when the same electrophysiological parameters were assessed in slices obtained from HFD-fed mice, we observed a complete ineffectiveness of leptin in modulating both spontaneous and evoked synaptic transmission. Indeed, leptin application under conditions identical to SD mice failed to modify mEPSCs and sEPSCs. The same was observed for evoked responses, i.e., AMPA EPSC amplitude, AMPA/NMDA ratio and PPF. These data show that HFD mice develop hippocampal resistance to leptin modulation of both spontaneous and evoked synaptic transmission.

Leptin exerts its cellular effects by triggering the activation of intracellular signaling cascades (Coppari and Bjørbæk, 2012). Thus, we reasoned that an analog of the functional resistance and insensitivity phenomena described above could exist at the molecular level. In order to establish a parallelism between synaptic activity and molecular pathways, we focused on STAT-3 signaling, the primary target of ObRb activation (Coppari and Bjørbæk, 2012). Leptin administration to SD mice stimulated ObRb phosphorylation and, with a longer latency, STAT-3 phosphorylation. This event has been shown to be required for both LTP in adult mice (Zearfoss et al., 2008) and LTD in juvenile rats (Nicolas et al., 2012), in agreement with the age-dependent polarity of leptin action on synaptic plasticity (Moult and Harvey, 2011). Moreover, this action does not require the nuclear translocation of STAT-3 (Nicolas et al., 2012), which agrees with the short latency of responses observed in electrophysiological experiments. On the contrary, HFD mice lost modulation of ObRb, STAT-3 and Akt phosphorylation in response to leptin. In addition, HFD mice displayed higher basal levels of hippocampal ObRb, STAT-3 and Akt activation, which can be correlated to the sustained increase in plasma leptin levels caused by HFD (Ahrén et al., 1997), and contributes to delineate the picture of leptin resistance. These findings are further confirmed by data from co-immunoprecipitation experiments, showing that HFD mice fail to exhibit the expected detachment of STAT-3 from ObR, which is a necessary step for successful signal transduction (Plum et al., 2006). The partial discrepancy of our data with those shown in Valladolid-Acebes et al. (2013) can be explained by the following factors: (i) use of a 45%-calories from fat diet (we used a 60%-calories from fat diet); (ii) shorter post-injection dissection time (90 min vs. 120 min); (iii) 1 mg/kg leptin dosage (we used 3 mg/kg); and (iv) no fasting before leptin injection (in our study, animals were fasted for 2 h before injection). In particular, fasting can be important to reset basal leptin levels (which can vary according to the satiety status of each animal) and could reduce baseline pSTAT-3 levels in SD-veh mice, thus unmasking the higher baseline pSTAT-3 in HFD-veh mice we observed. Finally, it is worth noting that increasing leptin dose can restore pSTAT-3 activation in HFD mice. This indicates that, at least in the 8-week HFD model, leptin resistance can be overcome. On the other hand, protracted HFD, can result, along with severe obesity, in loss of leptin responsiveness that cannot be restored by simply increasing the administered dose, also because of impaired transport across the blood-brain-barrier (Hileman et al., 2002).

One appealing explanation of the inability of HFD mice to initiate leptin-dependent signal transduction, despite higher baseline levels of ObRb phosphorylation, is increased activation of the negative feedback pathways impinging on this receptor. Among these, SOCS-3 plays a major role and has been suggested to be involved in leptin resistance (Balland and Cowley, 2015). Our data support this view, as we observed higher basal expression of SOCS-3 in HFD-fed mice, along with no modulation of its levels upon leptin stimulation. On the other hand, SD-fed mice responded to leptin with a pronounced increase in SOCS-3 levels. Results from co-IP experiments confirm this picture, with HFD mice displaying a higher basal association between SOCS-3 and ObR, which was not responsive to leptin stimulation. Vice versa, in SD mice, basal SOCS-3-ObR interaction was low and was dramatically increased by leptin administration.

Taken together, our results demonstrate that leptin stimulates neurotransmission, short-term plasticity of the SC-CA1 pathway, and activation of signal transduction pathways controlled by ObR. A hyperlypidic diet, however, blunts hippocampal signaling response to leptin and its functional and molecular consequences. Our findings also indicate that the hippocampus shares with the hypothalamus the leptin resistance caused by a prolonged exposure to HFD.

In addition to providing insight into the mechanisms of leptin action on the hippocampus and on their disruption in HFD, our work can be linked to the facts that metabolic alterations are now recognized as risk factors for Alzheimer’s disease (AD) (McGuire and Ishii, 2016) and that transgenic mouse models of AD display altered levels of circulating leptin, along with a reduction of its effects on hypothalamic neurons (Ishii et al., 2014). Thus, either leptin depletion or resistance can contribute to the neural plasticity deficits typical of AD. In this context, leptin treatment has been shown to ameliorate amyloid-β toxicity *in vitro* (Doherty et al., 2013) and behavioral deficits of transgenic models of AD (Greco et al., 2010). However, these works do not provide a strategy for overcoming defective plasticity and reduced cognitive performance in a condition of leptin resistance. Further research efforts will, therefore, be aimed at: (i) clarifying the molecular details of synaptic dysfunction caused by HFD; and (ii) finding strategies to restore hippocampal response to leptin.

## Author Contributions

MM conceived, designed and performed experiments, analyzed the data, wrote the manuscript; MS performed Western blot and co-IP experiments and analyzed the data; FS and MD contributed to electrophysiology experiments; AM contributed to Western blot experiments; SF set up the HFD model; CG designed experiments and revised the manuscript. All authors gave approval to the final version of the manuscript.

## Conflict of Interest Statement

The authors declare that the research was conducted in the absence of any commercial or financial relationships that could be construed as a potential conflict of interest.
