# Supplementary material for: Loss of Leptin-Induced Modulation of Hippocampal Synaptic Trasmission and Signal Transduction in High-Fat Diet-Fed Mice
Source: Front Cell Neurosci. 2017 Jul 28;11:225. doi: 10.3389/fncel.2017.00225 (PMC5532388; doi:10.3389/fncel.2017.00225)
Supplement: Supplementary file 1 [file Data_Sheet_1.pdf]

Supplementary Material

LOSS OF LEPTIN-INDUCED MODULATION OF HIPPOCAMPAL SYNAPTIC TRASMISSION AND SIGNAL TRANSDUCTION IN HIGH-FAT DIET-FED MICE

Marco Mainardi*, Matteo Spinelli, Federico Scala, Andrea Mattera, Salvatore Fusco, Marcello D'Ascenzo, and Claudio Grassi*.

Institute of Human Physiology, Università Cattolica Medical School, Largo F. Vito, 1 – 00168 Rome (ITALY)

*** Correspondence:** marco.mainardi@unicatt.it, claudio.grassi@unicatt.it

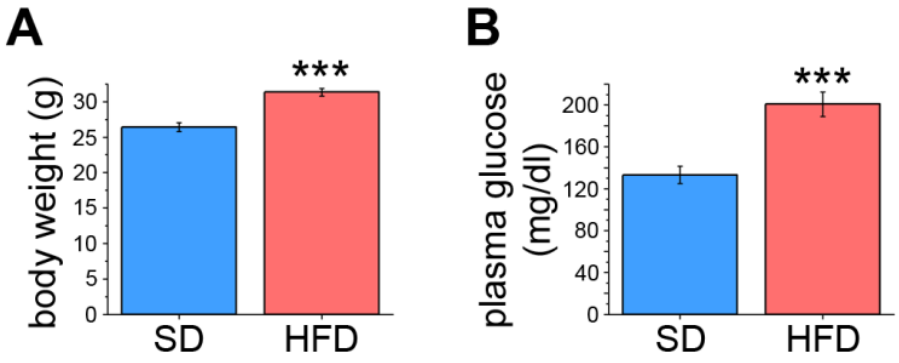

Supplementary Figure 1. Eight weeks of high-fat diet induce overweight and hyperglycemia in mice. A) Body weight of HFD mice is significantly higher than SD mice (SD $n = 20$, HFD $n = 21$; Student's t test, ***$p < 0.001$). B) Glycaemia of HFD mice is significantly higher than SD mice (SD $n = 10$, HFD $n = 10$; Student's t test, ***$p < 0.001$).

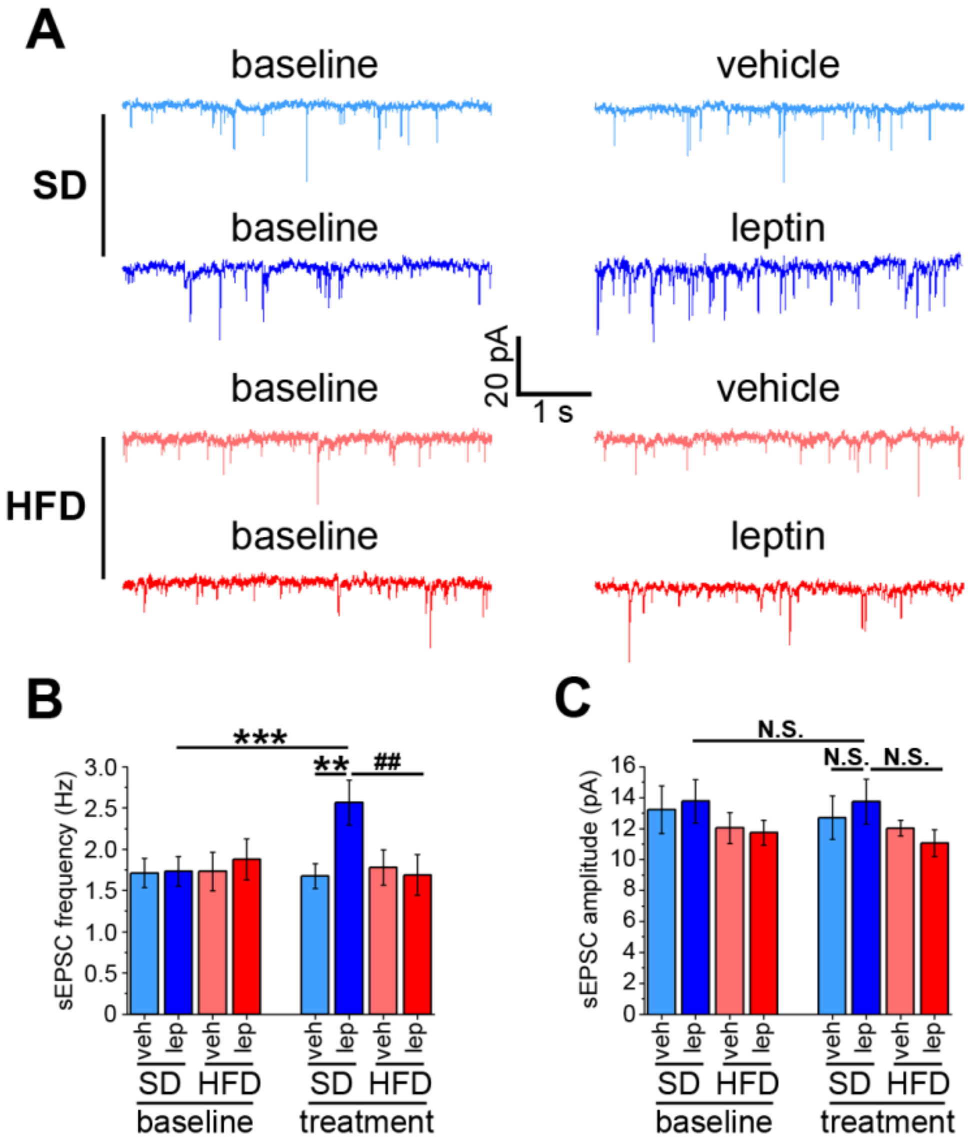

Supplementary Figure 2. Leptin modulates spontaneous neurotransmitter release in SD, but not in HFD, mice. A) Representative traces showing sEPSC recordings in SD and HFD mice treated with vehicle or leptin; both baseline and post-treatment (i.e., vehicle or leptin) recordings, corresponding to the same cell, are shown. B) sEPSC frequency is increased in SD mice in response to leptin application; in contrast, HFD mice are resistant to this effect (SD-veh $n = 11$, SD-lep $n = 13$, HFD-veh $n = 10$, HFD-lep $n = 10$; ANOVA-2 diet × treatment interaction, $p = 0.007$, followed by Bonferroni *post hoc* test, ***$p < 0.001$, **$p = 0.002$, $^{##}$p = 0.006). C) Leptin does not significantly affect the amplitude of sEPSCs in both SD and HFD mice (SD-veh $n = 11$, SD-lep $n = 13$, HFD-veh $n = 10$, HFD-lep $n = 10$; ANOVA-2, diet, $p = 0.523$, treatment, $p = 0.750$, diet × treatment interaction, $p = 0.989$).

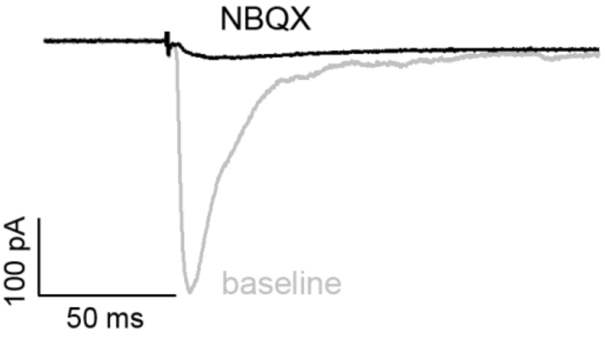

Supplementary Figure 3. The AMPA blocker NBQX abolishes EPSCs evoked on CA1 neurons by Schaffer Collateral stimulation. The baseline and the post-NBQX bath perfusion traces corresponding to the same cell are shown in gray and black, respectively.

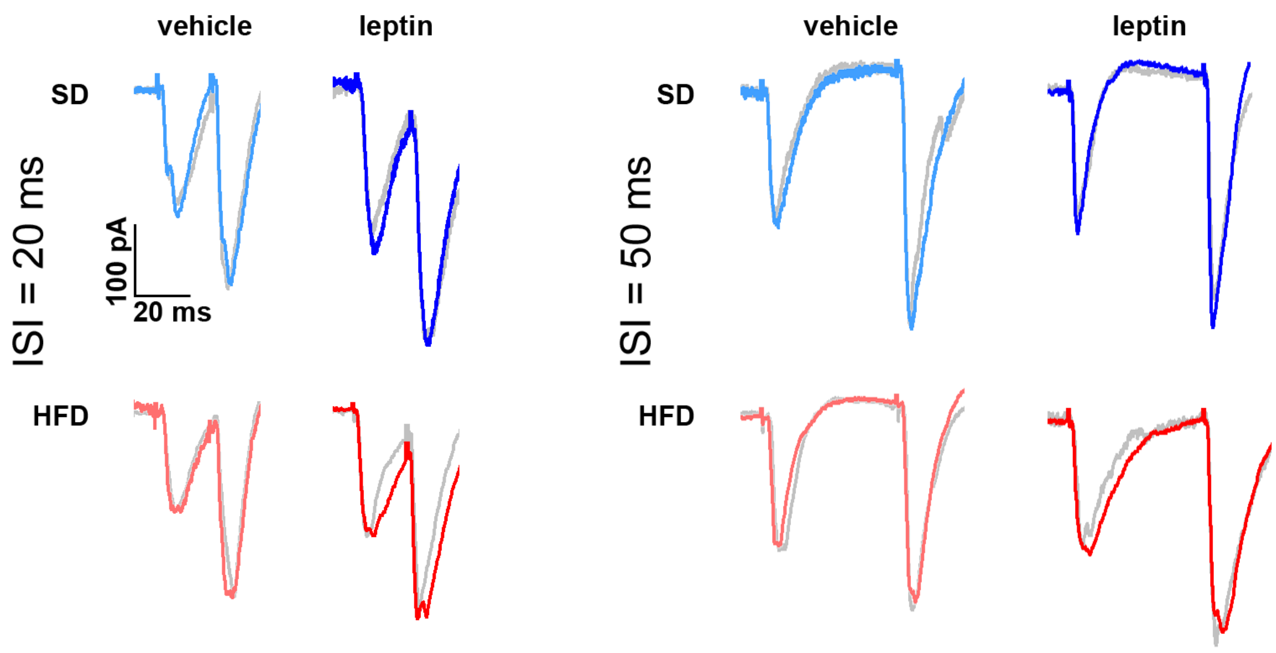

Supplementary Figure 4. Leptin does not affect paired-pulse facilitation (PPF) at short interstimulus intervals (ISIs). Representative traces showing EPSCs evoked by pair of stimuli delivered to the Schaffer collateral at 20 ms (left panel) and 50 ms (right panel) ISIs. Leptin has no effect on PPF at 20 ms ISI (SD-veh n = 10, SD-lep n = 10, HFD-veh n = 11, HFD-lep n = 14; ANOVA-2, diet, p = 0.635, treatment, p = 0.822, diet × treatment interaction, p = 0.945) and at 50 ms ISI (SD-veh n = 10, SD-lep n = 10, HFD-veh n = 11, HFD-lep n = 14; ANOVA-2, diet, p = 0.728, treatment, p = 0.898, diet × treatment interaction, p = 0.878).

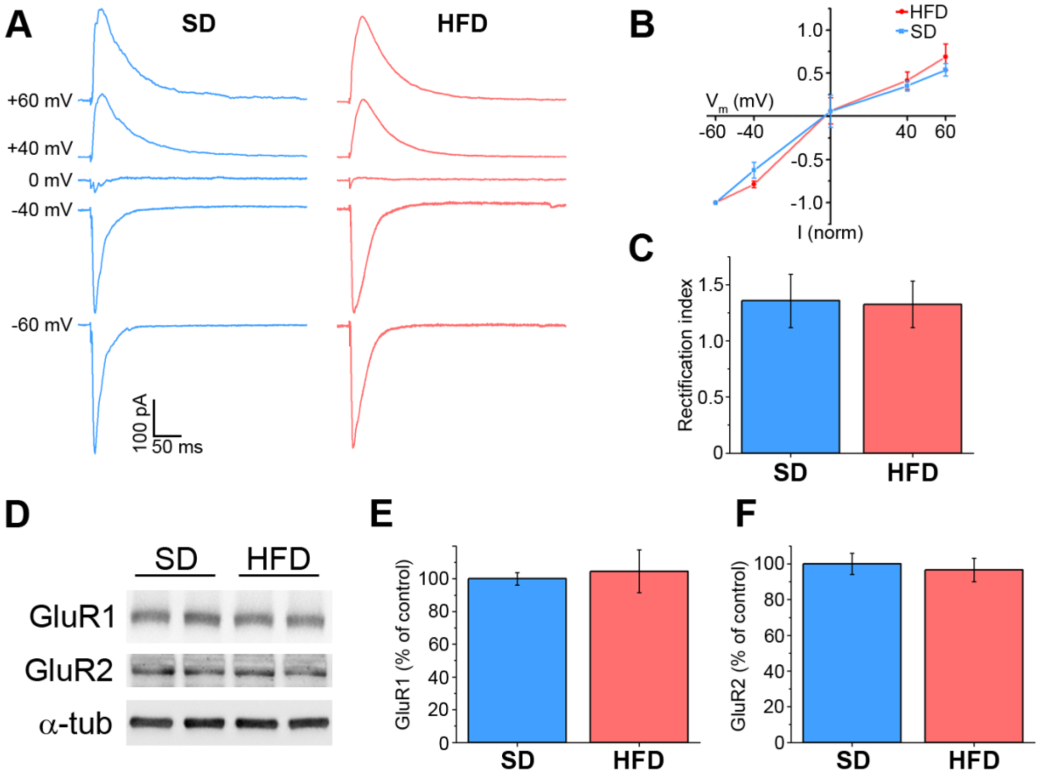

Supplementary Figure 5. SD and HFD mice display similar rectification indices for AMPA receptor-mediated EPSCs. A) Representative traces showing evoked EPSCs obtained from cells belonging to slices from SD and HFD (blue and red traces, respectively) in the presence of D-AP5 and PTX. B) I(V) curve for AMPA receptor-mediated EPSCs in SD and HFD and mice. C) Histogram showing the lack of statistically significant differences in the rectification indices of AMPA receptor-mediated EPSCs between SD and HFD mice (SD, n = 8, HFD, n = 9; Student's t test, p = 0.92). D) Representative images showing immunoblottings for GluR1, GluR2 and α-tubulin. E) GluR1 levels do not significantly differ between SD and HFD mice (SD n = 4, HFD n = 4; Student's t test, p = 0.75). F) GluR2 levels do not significantly differ between SD and HFD mice (SD n = 4, HFD n = 4; Student's t test, p = 0.72).

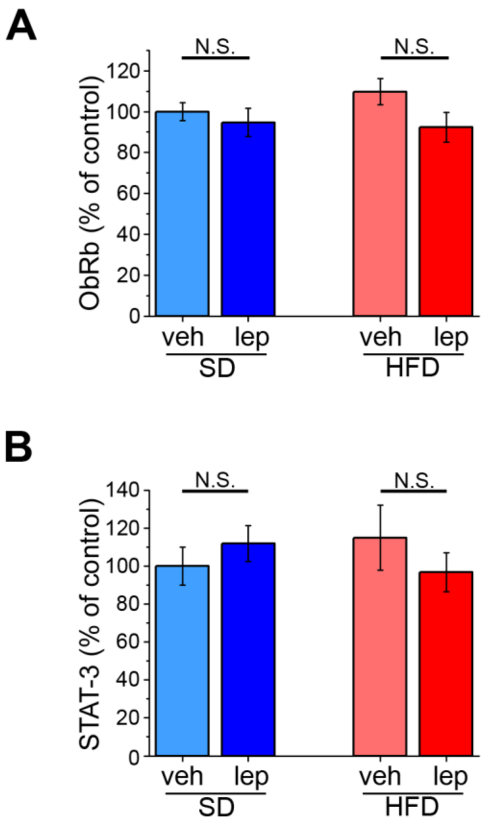

Supplementary Figure 6. Lack of significant differences in the expression levels of ObRb and STAT-3. A) Quantification of ObRb expression levels (SD-veh n = 4, SD-lep n = 4, HFD-veh n = 4, HFD-lep n = 4; ANOVA-2, diet, p = 0.574, treatment, p = 0.102, diet × treatment interaction, p = 0.361). B) Quantification of STAT-3 expression levels (SD-veh n = 5, SD-lep n = 6, HFD-veh n = 5, HFD-lep n = 5; ANOVA-2, diet, p = 0.995, treatment, p = 0.798, diet × treatment interaction, p = 0.225).

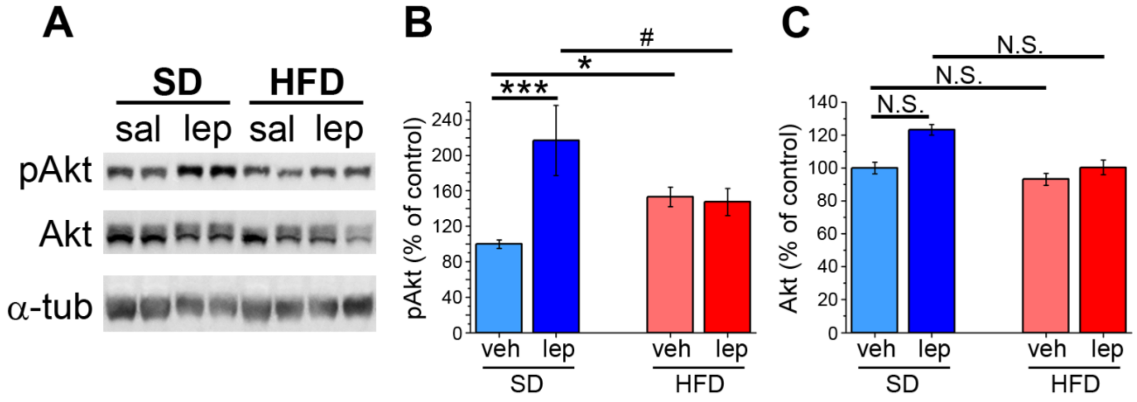

Supplementary Figure 7. Activation of Akt is impaired in HFD mice. A) Representative images showing immunoblottings for pAkt, Akt and α-tubulin. B) Quantification of pAkt activation (SD-veh n = 4, SD-lep n = 3, HFD-veh n = 4, HFD-lep n = 5; ANOVA-2, diet × treatment interaction, p = 0.004, followed by Bonferroni *post hoc* test, SD-lep vs. SD-veh, ***$p < 0.001$, SD-veh vs. HFD-veh, *p = 0.043, SD-lep vs. HFD-lep, $^{\#}p$ = 0.018). C) Quantification of Akt expression levels (SD-veh n = 4, SD-lep n = 3, HFD-veh n = 4, HFD-lep n = 5; ANOVA-2, diet, p = 0.08, treatment, p = 0.069, diet × treatment interaction, p = 0.704).

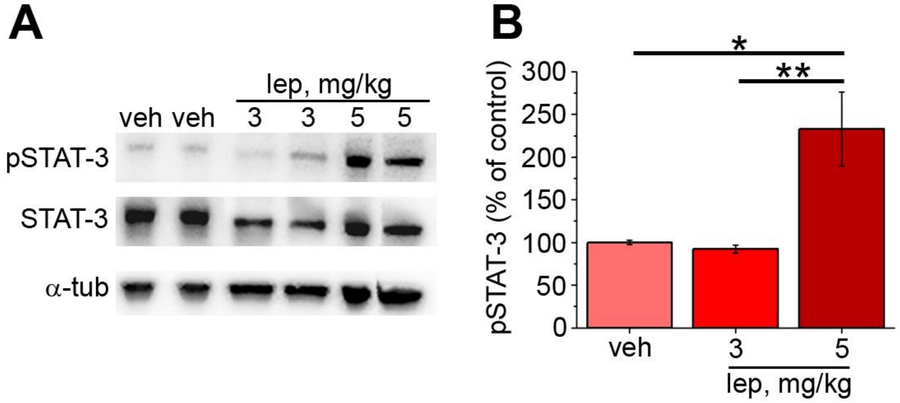

Supplementary Figure 8. Increasing leptin dosage can overcome blunted pSTAT-3 activation in HFD mice. A) Representative images showing immunoblottings for pSTAT-3, STAT-3 and α-tubulin. B) Quantification of pSTAT-3 activation (veh n = 3, lep-3mg/kg n = 4, lep-5mg/kg n = 3; ANOVA-1, p = 0.006, followed by Bonferroni *post hoc* test, *p = 0.016, **p = 0.008).
